# Effects of administration of co-trimoxazole and folic acid on sperm quality and histological changes of testes in male rats

**Published:** 2017-10

**Authors:** Ehsan Salarkia, Gholamreza Sepehri, Parvin Torabzadeh, Jalil Abshenas, Arezoo Saberi

**Affiliations:** 1 *Department of Biology, Karaj Branch, Islamic Azad University, Karaj, Iran.*; 2 *Kerman Neuroscience Research Center, Kerman University of Medical Sciences, Kerman, Iran.*; 3 *Department of Clinical Sciences, Faculty of Veterinary Medicine, Shahid Bahonar University of Kerman, Kerman, Iran.*; 4 *Kerman Physiology Research Center, Kerman University of Medical Sciences, Kerman, Iran.*

**Keywords:** Co-trimoxazole, Folic acid, Testis, Sperm quality, Rats

## Abstract

**Background:**

Male infertility has been reported following long-term sulfasalazine, however, the precise effects of co-trimoxazole on sperm quality is controversial.

**Objective:**

In this study, we evaluated the effects of co-trimoxazole and its co-administration with folic acid on sperm quality and histological changes of testes in male rats.

**Materials and Methods:**

In this experimental study, 136 male Wistar rats were divided into 9 groups: I (control), II (vehicle) received saline, III: received folic acid (1 mg/kg /daily i.p., and IV- IX received co-trimoxazole (30, 60, and 120 mg/kg/daily; i.p.)+folic acid (1 mg/kg/daily; i.p.) for 14 or 28 days. Sperm samples were obtained from each group at the end of 14^th^ and 28^th^ days. Sperm numbers, motility, and viability were evaluated on a hemocytometer. Hematoxylin and Eosin stained testes were done for evaluation ofthe number of Leydig cells, vascularity, spermatids, spermatocytes, and means of seminiferous tubules diameter under light microscopy.

**Results:**

Co-trimoxazole treatment for either 14 or 28 days caused a significant decrease in the percentage of sperm number, motility, and viability (p<0.001) compared to the control group. Also, high doses of co-trimoxazole caused a significant decrease in testes structural abnormalities means of seminiferous tubules diameter, spermatids, and spermatogonia) compared to the vehicle group (p<0.001). Folic acid co-administration with co-trimoxazole partially reversed the decrease in sperm quality and structural abnormalities of high doses of co-trimoxazole (60 and 120 mg/kg/daily) (p<0.001).

**Conclusion:**

The data showed the adverse effects of co-trimoxazole on sperm quality and testes morphology which was protected partially by folic acid co-administration in rats. The underlying mechanism (s) needs further investigations.

## Introduction

Several factors including social, genetic, and environmental factors may contribute to decline in the fertility rate of some couples (1). A male is said to be infertile if he is unable to impregnate his partner after one year of unprotected intercourse and in half of infertility cases, a male factor is involved (2). Approximately 10-15% of couples are infertile (3). 

Anatomical abnormalities such as varicocele may cause male infertility. Drugs-induced male infertility may be due to interfering of drugs with spermatogenesis, sperm motility, or by interfering with the fertilizing capacity of spermatozoa (4, 5). Co-trimoxazole, the combination of sulfamethoxazole and trimethoprim act by inhibition of a metabolic pathway for folic acid synthesis (6). The co-trimoxazole is used routinely to treat bacterial infections prior to in-vitro fertilization procedures. Long-term treatment with co-trimoxazole causes below-average semen parameters through a direct effect on sperm function and decrease in sperm count and impairs sperm motility characteristics (7). 

However, some studies have shown no difference in sperm quality of patients received 160 mg trimethoprim and 800 mg sulfamethoxazole twice a day as compared to control (8). It is reported that long-term sulfasalazine treatment (a sulfonamide drug used for the treatment of ulcerative colitis) caused abnormal semen quality (abnormal sperm motility and morphology) and low serum testosterone in men with inflammatory bowel disease (9). The precise mechanism (s) by which the sulfonamides affect the sperm quality may be mediated through the impairment of folic acid synthesis and intercalation with DNA (10).

Azizollahi *et al* showed that combination of zinc sulfate and folic acid treatment increased total normal sperm count in both subfertile and fertile men (11). Also, low paternal dietary folate alters the mouse sperm epigenome and is associated with negative pregnancy outcomes (12). It is reported that low folate in seminal plasma is associated with increased sperm DNA damage (13). It has shown that reactive oxygen species level is increased in the semen of 25-40% of infertile men and individuals with a low intake of antioxidant nutrients with a poor semen quality (14, 15). A systematic review of randomized studies showed the beneficial effects of oral antioxidants (vitamins C and E, zinc, selenium, folate, carnitine, and carotenoids) on sperm quality and pregnancy rate in infertile men (16).

Despite the possible adverse effects of co-trimoxazole on sperm quality, the drug is commonly used for the treatment of various infective conditions, however, there is no report on the effect of its co-administration with various antioxidants on sperm quality, so this study was designed to evaluate the effects of co-trimoxazole (30, 60, and 120 mg/kg/daily) and its combination with folic acid on sperm quality and histological changes of testes in male rats.

## Materials and methods

**Animals**

In this experimental study, 136 adult male Wistar rats weighing 200-250 gr were housed four/ cage (15×30×40 cm) in an air-conditioned animal house at 23±2^o^C on a 12 hr light/dark cycle with free access to the standard pellet and tap water. Before starting the experiment, the rats were allowed to accommodate to the laboratory environment for 1 wk (17).

**Experimental groups**

To investigate the effects of subacute administration of co-trimoxazole and its co-administration on sperm quality, all animal were sacrificed the day after 14th or 28th days after drug treatment. The animals were divided into 9 groups:

Group I: (Control) received no treatment (n=8).

Group II: (Vehicle) received daily intraperitoneal injection (i.p.) of normal saline for either 14 or 28 days (n=16).

Group III: received folic acid (1mg/kg/daily i.p.) for either 14 or 28 days (n=16).

Group IV to VI: received co-trimoxazole (30, 60, and 120 mg/kg/daily i.p.) for either 14 or 28 days (n=48).

Group VII to IX: received the combination of co-trimoxazole (30, 60, and 120 mg/kg/daily) and folic acid (1 mg/kg/daily) i.p.for either 14 or 28 days (n=48).

**Preparation of drug**

Co-trimoxazole tablets (400/80 mg, Sobhan Daru, Iran) and folic acid tablets (1 mg, Raha Daru, Iran) were crushed and suspended in 10 mL of normal saline to prepare a stock solution of both drugs. Then drugs were injected according to the above-mentioned protocol in experimental groups.

**Epididymal sperm parameters**

Rats were sacrificed by cervical dislocation at the end of each experiment and the testes were removed and fixed in 10% formalin for histological examinations. A small part of the cauda epididymis of each animal was dissected and located in 1 mL of pre-warmed Hams F10 medium (37^o^C, 5% CO_2_). Gentle tearing of the tissue was done to make spermatozoa swim out into the culture medium. The dishes were placed in the incubator for 15 min (18).

Sperm samples were obtained from each group at the end of 14^th^ and 28^th^ days. The mature sperm were collected from the caudate region of epididymis by a fine excision in phosphate buffer saline at 37^o^C and allowed to exude (15 min at 37^o^C, 5% CO_2_). Then, 1 ml distilled water was placed in a microtube and 20 ml of distilled water was replaced by sperm medium containing sperm cells. The following three parameters were used to determine the sperm quality: sperm concentration, motility and vitality. 

The sperm numbers were counted on a hemocytometer. Sperm suspensions from the caudal epididymis were diluted 1:200 with a fixative solution containing sodium acid carbonate and formaldehyde. Sperm cells were counted according to WHO laboratory manual for the examination of human semen and sperm. Cytological evaluation of sperm quality was carried out using a binocular microscope. The diluted samples were put into accounting chamber and the number of sperm was counted under a light microscope, using a haemocytometer with improved double Neubauer ruling and at least 400 spermatozoa were counted on each slide. 

The sperm concentration was expressed as ×10^6^/ml. For the determination of sperm motility, one drop of sperm suspension was placed on the slide and covered with a cover slip. Sperm motility was analyzed by the observation of motility of 100 sperm under the binocular light microscope using ×40 objective and averaged by counting the motile and non-motile spermatozoa and expressed as percentage. Eosin-nigrosin staining was used for the sperm vitality. One drop of sperm suspension was mixed with two drops of 1% eosin Y. After 30 sec, three drops of 10% nigrosin were added and mixed well. A smear was made by placing a drop of mixture on a clean glass slide and allowed to air dry and examine under oil immersion (1000×) with a binocular light microscope. 

Dead sperm cells were marked as pink and live sperm cells were marked as unstained. The sperm cells were counted under the light microscope and the sperm vitality was expressed as the percent of viable spermatozoa (19). Sperm suspension slide was stained and kept warm (37^o^C) in the incubator during the experiment. Aniline blue staining was used for assessing sperm maturity and light blue head were counted as mature sperm cells and heavy blue head as immature sperm cells (20, 21).

**Assessment of spermatozoa chromatin condensation**

The aniline blue staining was performed to evaluate sperm chromatin condensation. Briefly, after sperm preparation, 5 μl of the prepared spermatozoa were spread onto glass slides and allowed to dry. The smears were fixed in 3% buffered glutaraldehyde in 0.2 M phosphate buffer saline (pH=7.2) for 30 min. Slides were then stained with 5% aqueous aniline blue and mixed with 4% acetic acid (pH=3.5) for 5 min. About 100 sperm cells per slide were analyzed and the percentage of unstained sperm heads was calculated (22).

**Histopathological study**

For histological examination, testes were removed and weighted. Then, the testicular tissues were dissected and fixed in 10% neutral buffered formalin for microscopic examination at days 14 and 28. Formalin-fixed samples were processed by the standard paraffin wax technique. The paraffin sections were cut into 5 μm thick slices and stained with hematoxylin and eosin (H&E) and examined at 100 and 400× magnifications using a standard light microscope (22). 

The means of seminiferous tubules diameter was measured in each testis by Image Tools 2. The ten smallest, roundest tubules were identified for each animal per group and measured with an ocular micrometer under light microscopy. The number of Leydig cells vascularity, spermatids and spermatocytes were counted and compared to the control group. The other parameter was the percentage of spermatogenesis. For this purpose, 200 seminiferous tubules were examined under a light microscopy. The presence of spermatozoa within the seminiferous tubule was considered as the evidence of spermatogenesis (23, 24).

**Ethical consideration**

The experiments were conducted according to the guidelines on ethical standards for investigation of animals, which were approved by the Animal Experimentation Ethic Committee of Kerman Neuroscience Research Center (EC/KNRC/94/33).

**Statistical analysis**

All data were expressed as mean± standard error of at least 6 rats in each group. Statistical analysis was performed using one-way analysis of variance, followed by post hoc Tukey HSD test. A value of p<0.05 was considered statistically significant. Statistical analysis was performed using SPSS software (Statistical Package for the Social Sciences, version 20.0, SPSS Inc, Chicago, Illinois, USA).

## Results

**Effects of co-trimoxazole, folic acid, and their co-administration on sperm quality**

**Figure 1:**
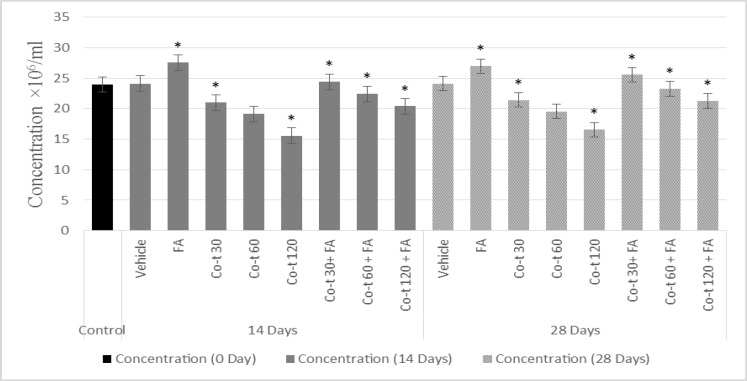
Effects of 14 and 28 days treatment with co-trimoxazole, folic acid and their co-administration on epidydymal sperm concentration in male rats.

**Figure 2:**
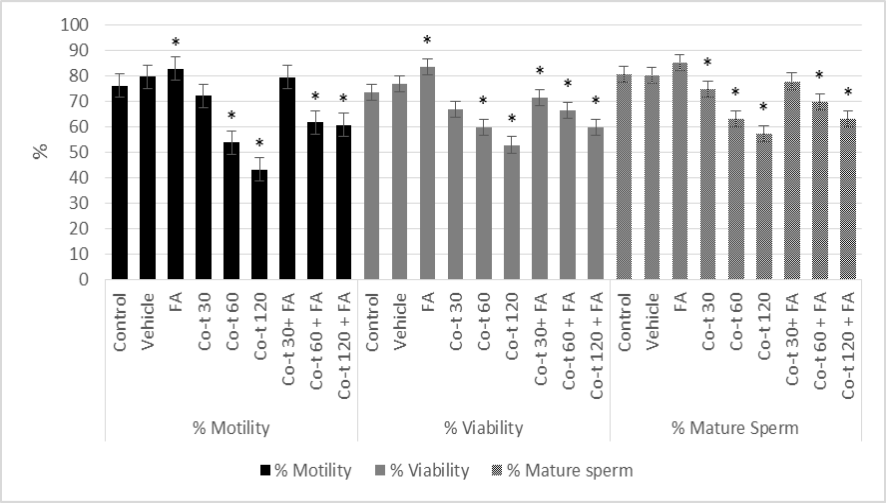
Effects of 14 days treatment with co-trimoxazole, folic acid and their co-administration on epididymal sperm motility, and viability in male rats

**Figure 3:**
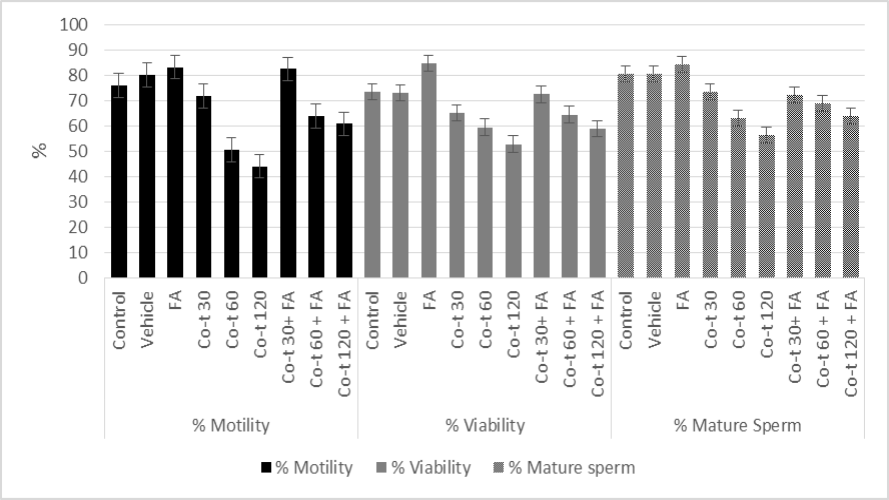
Effects of 28 days treatment with co-trimoxazole, folic acid and their co-administration on epididymal sperm motility, and viability in male rats.

Sperm parameters in rats assessed 14 and 28 days after co-trimoxazole, folic acid, and their combination exposure are shown in Figure 1. Our results showed that there were no significant changes in sperm count, motility, and percentage of normal morphology among the vehicle group compared to the control group (p=0.49, p=1.00) (Figure 2, 3). 

In group IV to VI, co-trimoxazole (30, 60, and 120 mg/kg/daily) for either 14 or 28 days was significantly associated, in a dose-dependent manner, with decrease in the percentage of sperm number. Also in this concentration, there was a significant decrease in the percentage of sperm motility (p<0.001) as well as sperm viability (p<0.001) compared with the control group (Figure 2, 3). Folic acid significantly increased the sperm number (p<0.001), sperm motility (p<0.001), and sperm viability (p<0.001) as compared to vehicle and control group. In group VII to IX, folic acid co-administration with co-trimoxazole treatment reversed the co-trimoxazole induced decrease in sperm quality (sperm number, motility, and viability), however, this combination partially, reversed the co-trimoxazole (60 mg/kg and 120 mg/kg) induced decrease in sperm quality, i.e. the sperm quality of co-administration of folic acid and co-trimoxazole was significantly lower than both control and vehicle group (p<0.001), (Figure 2, 3).

**Effects of co-trimoxazole, folic acid and their co-administration on testicular histopathology**

The mean testes weight in group IX were significantly lower than the control group (p=0.01). However, no significant decrease was observed in the other experimental groups compared to the control group (Table I).

Histological examination of the testes showed numerous structural changes in group VIII and IX compared to the control group. The main pathological changes included a significant decrease in means of seminiferous tubules diameter, primary and secondary spermatocytes, spermatogonia and Sertoli cells, compared to the control group. Co-trimoxazole had no effect on Leydig cell and testicular vascularity (Table II). No histological changes were seen in the control specimens (Figure 1). Folic acid significantly increased the sperm number (p<0.001), sperm motility (p<0.001) and sperm viability (p<0.001), and the number of spermatocytes, spermatogonia compared to vehicle and control group. In group VII to IX, increased the sperm number (p<0.001) sperm motility (p<0.001) and viability (p<0.001) as compared to group IV to VI (Table II). 

Also, folic acid reversed partially, not completely, the adverse effects of co-trimoxazole in group VIII and IX on the testes structure abnormalities including means of seminiferous tubules diameter, and the number of spermatids, spermatocytes and spermatogonia as compared to group V and VI, vehicle and control group (Table I, II). No significant changes in means of seminiferous tubules diameter, number of Leydig cells, Sertoli cells and testes vascularity were observed following folic acid treatment, however, its co-administration with co-trimoxazole significantly reversed the adverse effect of co-trimoxazole on means of seminiferous tubules diameter (p=0.02) and the number of Sertoli cells (p<0.001) compared to group IV to VI (Table II).

**Table I:** Effects of 14 and 28-day co-trimoxazole and folic acid administration compared with their co-administration on testes weight and structure in male rats

**Groups (n=8/each)**		**Testes weight (gr)**	**p-value** *	**Spermatogonia (n)**	**p-value ** *	**1** ^st^ ** Spermatocytes (n)**	**p-value** *	**2** ^nd ^ **Spermatocytes (n)**	**p-value** *	**Presence of spermatozoa (%)**	**p-value** *
Control		1.68 ± 0.05	---	50.13 ± 0.29	---	51.33 ± 0.30	---	50.98 ± 0.24	---	85.30 ± 1.50	---
14 Days											
	Vehicle	1.58 ± 0.05	0.99	50.48 ± 0.26	1.00	51.37 ± 0.27	1.00	50.97 ± 0.24	1.00	89.23 ± 0.60	1.00
	FA	1.79 ± 0.05	0.98	52.80 ± 0.22	<0.001	52.57 ± 0.19	0.14	52.39 ± 0.18	0.01	86.32± 1.15	1.00
	Co-t 30	1.57 ± 0.04	0.97	49.36 ± 0.20	0.97	49.92 ± 0.19	0.04	50.06 ± 0.19	0.37	80.33 ± 0.68	0.93
	Co-t 30 + FA	1.55 ± 0.05	0.91	50.45 ± 0.23	1.00	51.40 ± 0.22	1.00	51.07 ± 0.19	1.00	79.59 ± 0.51	0.84
	Co-t 60	1.55 ± 0.04	0.89	47.45 ± 0.33	<0.001	46.20 ± 0.23	<0.001	44.48 ± 0.29	0.001	65.21 ± 1.13	<0.001
	Co-t 60 + FA	1.47 ± 0.08	0.21	49.24 ± 0.26	0.90	49.95 ± 0.21	0.05	48.43 ± 0.26	0.001	70.23 ± 0.48	<0.001
	Co-t 120	1.52 ± 0.03	0.63	41.00 ± 0.27	<0.001	40.98 ± 0.39	<0.001	40.58 ± 0.25	0.001	53.29 ± 0.68	<0.001
	Co-t 120 + FA	1.50 ± 0.04	0.41	44.44 ± 0.40	<0.001	43.29 ± 0.25	<0.001	42.61 ± 0.20	0.001	69.73 ± 0.53	<0.001
28 Days											
	Vehicle	1.65 ± 0.03	1.00	49.32 ± 0.37	0.98	51.72 ± 0.25	1.00	49.68 ± 0.32	0.05	85.33 ± 1.45	1.00
	FA	1.79 ± 0.03	0.97	52.81 ± 0.20	<0.001	52.74 ± 0.21	0.04	52.45 ± 0.18	0.001	92.33 ± 0.88	1.00
	Co-t 30	1.59 ± 0.07	0.99	49.17 ± 0.20	0.83	49.60 ± 0.16	1.00	49.56 ± 0.16	0.001	80.36 ± 0.67	0.92
	Co-t 30 + FA	1.52 ± 0.06	0.63	50.63 ± 0.26	1.00	51.69 ± 0.24	1.00	51.06 ± 0.20	1.00	81.23 ± 0.67	<0.001
	Co-t 60	1.57 ± 0.06	0.97	45.90 ± 0.61	<0.001	46.19 ± 0.22	<0.001	44.26 ± 0.26	0.001	72.25 ± 0.33	<0.001
	Co-t 60 + FA	1.68 ± 0.04	1.00	48.99 ± 0.26	0.57	49.63 ± 0.21	<0.001	48.68 ± 0.25	0.001	78.32 ± 0.58	<0.001
	Co-t 120	1.39 ± 0.05	0.01	40.02 ± 0.37	<0.001	40.48 ± 0.44	<0.001	40.44 ± 0.25	0.001	64.23 ± 0.88	<0.001
	Co-t 120 + FA	1.50 ± 0.03	0.47	43.57 ± 0.23	<0.001	43.10 ± 0.26	<0.001	42.75 ± 0.20	0.001	75.00 ± 0.58	<0.001

Rats received co-trimoxazole (30, 60 and 120 mg/kg/daily; i.p.), folic acid (1 mg/kg/daily; i.p) or their combination for either 14 or 28 days. Vehicle group received saline. Control rats received no treatment. All data presented as Mean ± SEM.

Co-t: Co-trimoxazole

FA: Folic acid

* Compared with control group

**Table II:** Effects of 14 and 28-day co-trimoxazole and folic acid administration compared with their co-administration on testes vascularity, MSTD, Leydig cells, and Sertoli cells in male rats

**Groups (n=8/each)**		**MSTD (µm)**	**p-value ** *	**Leydig cell (n)**	**p-value** *	**Sertoli cell (n)**	**p-value** *	**Vascularity %**	**p-value** *
Control		299.67 ± 7.74	---	10.42 ± 0.24	---	10.40 ± 0.22	---	1.75 ± 0.22	---
14 Days									
	Vehicle	314.83 ± 7.60	0.61	10.62 ± 0.24	1.00	10.20 ± 0.19	1.00	1.83 ± 0.21	1.00
	FA	301.00 ± 6.00	1.00	10.76 ± 0.17	1.00	11.02 ± 0.17	0.59	1.83 ± 0.21	1.00
	Co-t 30	279.50 ± 3.44	<0.001	10.30 ± 0.18	1.00	10.01 ± 0.18	1.00	1.67 ± 0.22	1.00
	Co-t 30 + FA	285.33 ± 3.57	0.71	10.48 ± 0.19	1.00	10.31 ± 0.18	1.00	1.50 ± 0.19	1.00
	Co-t 60	274.83 ± 6.17	0.02	10.05 ± 0.15	1.00	8.98 ± 0.15	<0.001	1.58 ± 0.19	1.00
	Co-t 60 + FA	289.50 ± 3.93	0.98	10.14 ± 0.22	1.00	10.33 ± 0.16	1.00	1.75 ± 0.22	1.00
	Co-t 120	275.50 ± 4.56	0.03	10.14 ± 0.18	1.00	8.39 ± 0.16	<0.001	1.67 ± 0.22	1.00
	Co-t 120 + FA	287.17 ± 1.30	0.87	10.20 ± 0.19	1.00	9.54 ± 0.16	0.44	1.75 ± 0.18	1.00
28 Days									
	Vehicle	307.17 ± 2.58	1.00	10.40 ± 0.24	1.00	10.37 ± 0.22	1.00	1.83 ± 0.21	1.00
	FA	302.33 ± 4.58	1.00	11.31 ± 0.19	0.16	11.01 ± 0.17	0.55	1.83 ± 0.21	1.00
	Co-t 30	280.33 ± 1.80	1.00	10.23 ± 0.18	0.16	10.19 ± 0.19	0.99	1.67 ± 0.22	1.00
	Co-t 30 + FA	286.83 ± 1.79	0.71	10.46 ± 0.20	1.00	10.60 ± 0.19	1.00	1.50 ± 0.19	1.00
	Co-t 60	262.83 ± 6.14	<0.001	10.11 ± 0.19	1.00	8.51 ± 0.13	<0.001	1.58 ± 0.19	1.00
	Co-t 60 + FA	297.33 ± 3.13	1.00	10.32 ± 0.23	1.00	10.39 ± 0.15	1.00	1.75 ± 0.22	1.00
	Co-t 120	254.00 ± 1.12	<0.001	10.10 ± 0.20	1.00	8.52 ± 0.15	<0.001	1.67 ± 0.22	1.00
	Co-t 120 + FA	274.83 ± 2.33	0.02	10.20 ± 0.17	1.00	9.74 ± 0.15	0.07	1.75 ± 0.18	1.00

Rats were received co-trimoxazole (30, 60 and 120 mg/kg/daily, i.p), folic acid (1 mg/kg/daily, i.p.) or their combination for either 14 or 28 days. Vehicle group received saline. Control rats received no treatment. All Data presented as the Mean ± SEM.MSTD: means of seminiferous tubules diameter,

Co-t: Co-trimoxazole

FA: Folic acid

* Compared with control group

Rats were received co-trimoxazole (30, 60 and 120 mg/kg), folic acid (1 mg/kg) or their combination intraperitoneally for either 14 or 28 days

Folic acid treatment, significantly reversed the adverse effect of co-trimoxazole on epidydymal sperm concentration compared to co-trimoxazole group. Vehicle group received saline. Control rats received no treatment. Data are the Mean ± SEM of 8 rats in each group.

Co-t= Co-trimoxazole

FA= Folic acid

* Compared to control group

Rats were received co-trimoxazole (30, 60 and 120 mg/kg), folic acid (1 mg/kg) or their combination intraperitoneally for 14 days. Folic acid treatment, significantly reversed the adverse effect of co-trimoxazole on epididymal sperm motility, and viability in male rats.Vehicle group received saline. Control rats received no treatment. Data are the Mean ± SEM of 8 rats in each group.

Co-t= Co-trimoxazole

FA= Folic acid

* Compared to control group

Rats were received co-trimoxazole (30, 60 and 120 mg/kg), folic acid (1 mg/kg) or their combination intraperitoneally for 28 days. Folic acid treatment, significantly reversed the adverse effect of co-trimoxazole on epididymal sperm motility, and viability in male rats.Vehicle group received saline. Control rats received no treatment. Data are the Mean ± SEM of 8 rats in each group.

Co-t= Co-trimoxazole

FA= Folic acid

* Compared with control group

**Figure 4:**
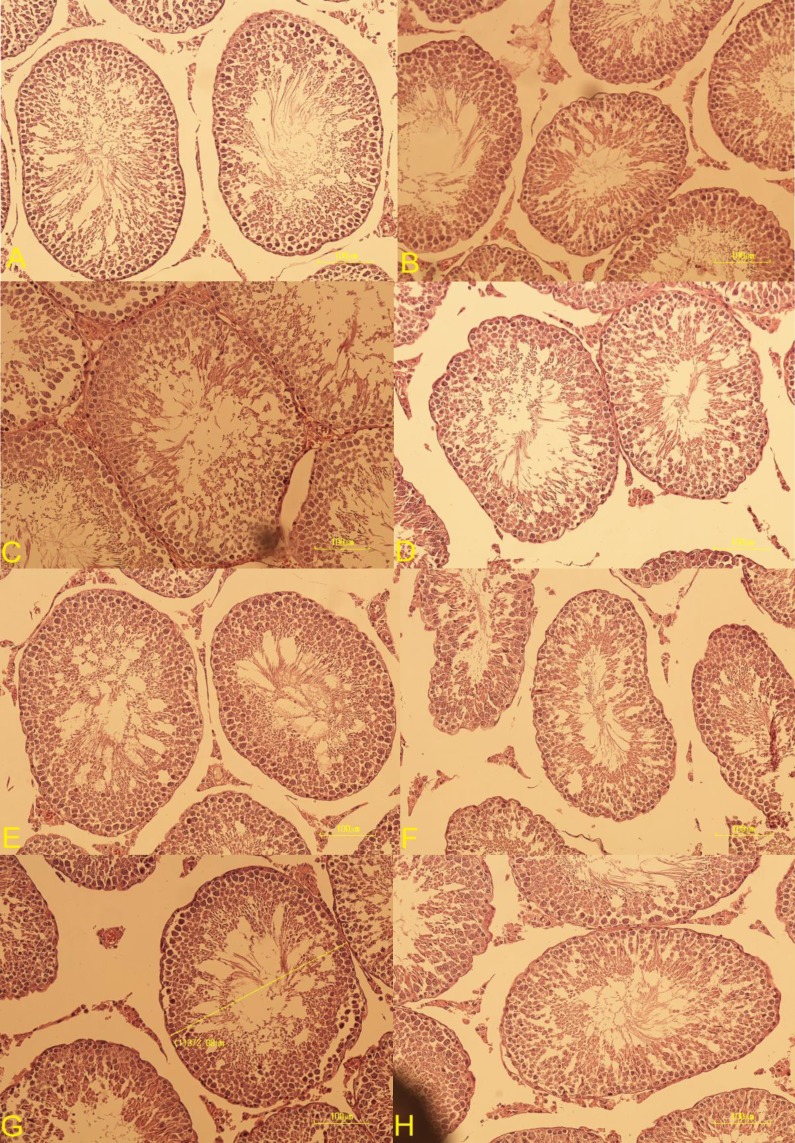
Histological images of testeshematoxylin and eosin (H&E) staining (×400) in studid groups (A-H)

(A) Control group received no treatment, (B) Folic Acid 1mg/kg/daily, (C) Co-trimoxazole 30 mg/kg, (D) Co-trimoxazole 30 mg/kg/daily + Folic Acid 1mg/kg, (E) Co-trimoxazole 60 mg/kg, (F) Co-trimoxazole 60 mg/kg/daily + Folic Acid 1mg/kg, (G) Co-trimoxazole 120 mg/kg/daily, (H) Co-trimoxazole 120 mg/kg/daily + Folic Acid 1mg/kg/daily.

No histological changes were seen in the control group, folic acid alone did not show any significant effects on testes structure.

High dose of co-trimoxazole (120 mg/kg) showed adverse effects on testes structure, Folic acid co-administration with co-trimoxazole reversed these effects partially, but not completely

## Discussion

The results of present study indicated that co-trimoxazole at a dose of 60 and 120 mg/kg for either 14 or 28 days caused a significant decrease in sperm count, viability, progressive motility and normal morphology in all exposure groups compared with the control group. Also, it caused structural abnormalities indicated a significant decrease in means of seminiferous tubules diameter, and a number of spermatocytes, spermatids and spermatogonia, 14 or 28 days post co-trimoxazole treatment.

These results are in agreement with previous reports indicated signiﬁcant impairment of spermatogenesis by co-trimoxazole treatment through interfering with folic acid synthesis and metabolism in rats (24). Sulfasalazine which is used for many years as a treatment for ulcerative colitis or Crohn’s disease has been associated with oligospermia, a decrease in sperm motility and male infertility (25). High in vitro concentrations (5 mg/ml) of Sulfamethoxazole alone signiﬁcantly reduced progressive motility of human spermatozoa and its combination with trimethoprim increased the sensitivity of spermatozoa to the drug approximately 10-fold (26).

Hargreaves *et al* showed that sperm viability was signiﬁcantly reduced following in vitro exposure to co-trimoxazole, erythromycin, amoxicillin and tetracycline (10). The effect of co-trimoxazole on semen parameters and human fertility is controversial. Lange and Schirren showed a signiﬁcant adverse effect on all semen parameters (motility, morphology and count) following four weeks treatment with co-trimoxazole (22). In contrast, another study showed no significant alteration in semen parameters following treatment for one month with co-trimoxazole in men with bacteriological-positive culture (8).

Co-trimoxazole induced alterations in semen parameters could be due to inhibition of folate synthesis which result in low semen quality (24). Also, it could be related to either the presence or the eradication of the infection which affects the sperm quality following semen bacterial contamination (10, 27).

Our results showed that folic acid alone increased the semen quality and also its co-administration with co-trimoxazole reversed the adverse effects of high dose of co-trimoxazole (60 and 120 mg/kg) on sperm quality through the increasing the sperm concentration, motility and viability and amelioration of testes structural abnormalities by increasing means of seminiferous tubules diameter, and number of spermatids, spermatocyte and spermatogonia. Our results are in complete agreement with previous reports indicating that folic acid intervention treatment significantly increased sperm concentration in subfertile males (28). The increase in sperm concentration after the folic acid intervention was not the result of alterations in FSH, testosterone or inhibin B concentrations (28). Men with high folate intake had lower overall frequencies of several types of euploid (presence of an abnormal number of chromosomes) sperm (29). Folic acid is necessary for the de novo synthesis of purines and thymidylate and DNA synthesis (30). Therefore, in somatic cells, folate deﬁciency can lead to increased uracil incorporation into DNA, DNA double-strand breaks, genome instability and DNA hypomethylation and hence the DNA damage and or altered DNA and histone methylation in germ cells could lead to impair spermatogenesis and decreas the sperm counts (30, 31). Since folate is involved in the synthesis of DNA and RNA, so folate deficiency may lead to decrease spermatogenesis and impaire male fertility and low folate in seminal plasma is correlated with decreased sperm counts and increased sperm DNA damage in humans (32, 33). Therefore, it is proposed that adequate folic acid intake in adulthood could be important for preventing chromatin damage and mutation in the male germ line. Although folate has several effects on spermatogenesis, the underlying mechanisms involved are not clear and need further investigations (28).

## Conclusion

The data show the adverse effects of co-trimoxazole on sperm quality (sperm number, motility and viability) and testes morphology indicating a significant decrease in means of seminiferous tubules diameter, the number of spermatids, spermatogonia and spermatocytes in adult male rats. Folic acid significantly increased the sperm quality and partially reversed the co-trimoxazole induced a decrease in sperm quality and testes structural abnormalities in rats. The underlying mechanism(s) is not known yet and needs further investigation.

## Note

***This article extracted from M.Sc. thesis. (Ehsan Salarkia)***
